# A review of technologies for collaborative online information seeking: On the contribution of collaborative argumentation

**DOI:** 10.1007/s10639-020-10345-7

**Published:** 2020-09-30

**Authors:** Elisabeth Mayweg-Paus, Maria Zimmermann, Nguyen-Thinh Le, Niels Pinkwart

**Affiliations:** 1 Einstein Center Digital Future | Department of Education Studies | Digital Knowledge Management in Higher Education, Humboldt University of Berlin, Unter den Linden 6, 10099 Berlin, Germany; 2 Department of Computer Science | Research Group Computer Science Education | Computer Science and Society, Humboldt University of Berlin, Unter den Linden 6, 10099 Berlin, Germany

**Keywords:** Collaborative information seeking technologies, Computer-supported collaborative learning and argumentation, Critical thinking, Sourcing of online information

## Abstract

In everyday life, people seek, evaluate, and use online sources to underpin opinions and make decisions. While education must promote the skills people need to critically question the sourcing of online information, it is important, more generally, to understand how to successfully promote the acquisition of any skills related to seeking online information. This review outlines technologies that aim to support users when they collaboratively seek online information. Upon integrating psychological–pedagogical approaches on trust in and the sourcing of online information, argumentation, and computer-supported collaborative learning, we reviewed the literature (*N* = 95 journal articles) on technologies for collaborative online information seeking. The technologies we identified either addressed collaborative online information seeking as an exclusive process for searching for online information or, alternatively, addressed online information seeking within the context of a more complex learning process. Our review was driven by three main research questions: We aimed to understand whether and how the studies considered 1) the role of trust and critical questioning in the sourcing of online information, 2) the learning processes at play when information seekers engage in collaborative argumentation, and 3) what affordances are offered by technologies that support users’ collaborative seeking of online information. The reviewed articles that focused exclusively on technologies for seeking online information primarily addressed aspects of cooperation (e.g., task management), whereas articles that focused on technologies for integrating the processes of information seeking into the entire learning processes instead highlighted aspects of collaborative argumentation (e.g., exchange of multiple perspectives and critical questioning in argumentation). Seven of the articles referred to trust as an aspect of seekers’ sourcing strategies. We emphasize how researchers’, users’, and technology developers’ consideration of collaborative argumentation could expand the benefits of technological support for seeking online information.

## Introduction

Seeking information on the Web is a prevalent and preferred way to obtain information and acquire knowledge about any issue related to one’s personal or professional life (e.g., Cash et al. 2014; Purdyet et al. 2015). However, people can only seek online information effectively and derive justified decisions based to their own knowledge boundaries and skills related to finding, evaluating, and using information of good quality (i.e., complete, accurate, and appropriate information) (Bromme and Goldman 2014). Being able to deal competently with information online becomes important, for example, as people seek social and scientific information on the COVID-19 pandemic.

Yet, because information seekers generally have difficulty seeking online information effectively (e.g., Chen et al. 2019; Iding et al. 2008; Metzger and Flanagin 2013; Nygren and Guath 2019; Rieh et al. 2012; Tsai et al. 2012), education systems have been tasked with promoting students’ skills and competencies in this area. As students should be taught to be more competent with and within an evolving digitalization of information societies (e.g., Caena and Redecker 2019; Ferrari 2013), many researchers have been trying to understand what skills this may entail and how these skills can be promoted, such as through information literacy, science education, or computer-supported collaborative learning (CSCL) (e.g., Forte 2015; Solli et al. 2018; Tabak 2015). The COVID-19 pandemic, as a social event, is not only requiring us to be able to competently search for online information, but it is also forcing us to have almost every social interaction virtually, to minimize contagion. Over the last few years, the ability for users to collaboratively seek online information has been supported with the emergence of several new technologies (e.g., Hertzum and Hansen 2019).

This review aims to identify the educational potential of technologies that support collaborative online information seeking by focusing on the role of collaborative argumentation within the process of sourcing online information. In this sense, to outline the importance of distinct argumentative behavior such as critical questioning for the sourcing of information, we introduce psychological–pedagogical approaches on *sourcing online information* and *computer-supported collaborative learning*. Further, to investigate how and to what extent collaborative argumentation is addressed in the literature, we review articles on technologies that support the collaborative seeking of online information.

## Seeking online information

When seeking online information collaboratively, how might theoretical approaches and concepts help us? First, since many concepts related to the phenomenon of seeking online information (either individually or collaboratively) are accompanied by diverse terms (e.g., (collective) information retrieval, (collaborative) information search, (collective) information retrieval, (collaborative) information behavior, or (collaborative) knowledge building) (see also Gonzalez-Teruel et al. 2015; Shah 2014; Shah et al. 2017), the specific meaning of *seeking information* might encompass an individual browsing the Web for health information (Pian et al. 2016), another individual finding, evaluating, and using science-related online information (Bromme and Goldman 2014), multiple researchers working together collaboratively in academic research (Farooq et al. 2009), as well as an individual consulting Wikipedia, which represents the collaborative outcome of learning processes (e.g., Alterman and Harsch 2017). In this sense, psychological–pedagogical research often refers to *seeking online information* as any seeking that is accompanied by active and passive acts of searching, finding, selecting, evaluating, and synthesizing (or using) information; it includes any attempt a seeker makes to acquire knowledge about a topic of interest (e.g., Bromme and Goldman 2014; Nauroth et al. 2015; Reddy et al. 2018; Wilson 2000).

These actions that information seekers perform are more broadly referred to as *online information behavior*, and they arise due to the interplay of the information seeker’s psychological features, the content, and the media; together, these components (e.g., one’s searching skills, one’s motivation, the comprehensibility of information, and the media’s ease of use) arouse information seekers’ to action toward a desired goal (e.g., Bromme and Goldman 2014; Nauroth et al. 2015; Reddy et al. 2018; Wilson 2000).

While some theories on online information sourcing exclusively focus on the processes of searching for online information (e.g., Kuhlthau’s Information Search Process (ISP) model: Kuhlthau 1993), other theories consider the processes of searching for online information as a part of a broader learning process that involves searching, evaluating, and using online information (e.g., Risk Information Search and Processing Model (RISP): Griffin et al. 1999; Multiple Document Trace Model: e.g., Rouet and Britt 2011; Cognitive Affective Engagement Model (CAEM): e.g., List and Alexander 2017). Similarly, technologies may focus on how to support just the searching for information (e.g., with functions that allow users to share search results) or on how to support users with the learning that occurs during the searching (e.g., with functions that allow users to discuss their search results).

## The role of trust when sourcing online information

*Trust* plays a crucial role in psychological–pedagogical research on sourcing online information (e.g., Aljazzaf et al. 2010; Bromme and Goldman 2014; Jones and Moncur 2018; Sillence et al. 2006), in which the focus is often on information seekers’ perception of and strategies for evaluating whether they can rely on the online information (e.g., Bromme and Goldman 2014; Hargittai et al. 2010; Wathen and Burkell 2002; Zimmermann and Jucks 2018a). In this sense, research has identified certain criteria that seem to impact information seekers when evaluating the trustworthiness of information, providers of information, and media; these criteria are the scientific nature of the information, the information providers’ expertise, and the navigability of media (e.g., Choi and Stvilia 2015; Hilligos and Rieh, 2008; Metzger and Flanagin, 2013; Sundar 2008; Thomm and Bromme 2016; Zimmermann and Jucks 2018a, b).

Building upon approaches on systematic and heuristic information processing (Chaiken 1980; Cacioppo et al. 1986), we assume that, depending on an information seeker’s capacity, motivation, and relevance, their evaluation of online information takes place rather systematically or heuristically. With respect to the effort it takes to evaluate online information systematically, seekers seem to know that they tend to use both relevant criteria (e.g., whether content entails plausible arguments and references) and irrelevant criteria (e.g., layout of website, rank of search engine for evaluating online information) (Eysenbach and Köhler 2002; Metzger et al. 2010). However, it is challenging for information seekers to come up with better strategies for evaluating the quality of online information, as even highly knowledgeable and motivated information seekers who aim to systematically evaluate the correctness, completeness, and appropriateness of information eventually reach the limits of their own knowledge and capacity (e.g., it is likely that a highly knowledgeable engineer is incompetent in another field of expertise).

As an indirect way to determine whether online information is trustworthy, information seekers may use hints about whether the provider of the online information seems trustworthy (e.g., whether she uses jargon). This strategy may be effective and efficient, but only when proper hints are used and the seeker’s information needs are satisfied in a timely and efficient way (Bromme and Goldman 2014; Rieh et al. 2016). In this sense, a crucial factor for seeking online information successfully is that the information seeker needs to critically reflect on their seeking behavior and sourcing strategies (e.g., Bråten et al. 2019; Forte 2015; Rieh et al. 2016; Pérez et al. 2018; second author and first author submitted). While students in a training on sourcing online information did initially use and check for information about the trustworthiness of the provider of information, they did not critically reflect on what they found from the trustworthiness check (Brante 2019). However, in educational interventions that encouraged students to not only identify and assess trustworthiness criteria but also critically reflect on the sourcing of information, students more elaborately integrated information about the perceived trustworthiness of the provider (Bråten et al. 2019; Pérez et al. 2018).

Although these trainings focused on promoting individual students’ sourcing competencies, it is possible that integrating elements of collaborative argumentation during the sourcing process might stimulate “additional” and “deeper” critical reflection as well as more elaborated reasoning (second author and first author submitted). Building on the potential of reasoning activities, collaborative argumentation should play a central role within information seeking and source evaluation processes, as it may not only facilitate more successful engagement with the information, but it may also promote explicit and critical questioning of the sourcing of information as well as the trustworthiness of information providers and media in online contexts (e.g., Bråten et al. 2019; Pérez et al. 2018; second author and first author submitted). Based on these considerations, in the following we outline approaches on computer-mediated collaborative learning to emphasize how collaboration from a psychological–pedagogical perspective may contribute to seeking online information successfully.

## Cooperation and collaboration in seeking online information

Research findings within the last decades have suggested that learning with others may strongly benefit knowledge gains (Hattie 2009; Kyndt et al. 2013) as well as affective, motivational, and social variables (Springer et al. 1999). A recent meta-analysis revealed that collaborative learning positively affects knowledge acquisition, student perceptions, and skill development in digital learning contexts (e.g., Chen et al. 2018). Given these findings, collaboration with others is likely to show advantages also in the context of seeking online information.

To transfer and adjust our knowledge about the benefits of collaborative learning to the specific field of seeking online information, we would need to take a deeper look at the conceptualizations and mechanisms involved in collaborative learning. In this regard, it seems promising to start with further clarification of what *collaboration* in learning means in contrast to *cooperation* (Baker 2015; Siemon et al. 2019), terms that are often used interchangeably but do not mean the same thing, and in the context of seeking online information, have different educational implications. Following the initial definition by Roschelle and Teasley (1995), collaboration always addresses “the mutual engagement of participants in a coordinated effort to solve a problem together” (p.70), thereby focusing on the processes of group members or learners working together. In contrast, in cooperation, learners usually define subtasks that they work on individually, although they might share a common focus. Based on this, Baker (2015) provides the following definition: “Collaboration is a specific form of cooperation: cooperation works on the level of tasks and actions, collaboration works on the plane of ideas, understanding, representations (p.5)”.

Whether cooperation or collaboration is beneficial in terms of a desired outcome in the context of seeking information should depend on the goal, which might differ within different stages of the information seeking process (i.e., finding, evaluating, and using online information), and might also depend on information seeking process in general (e.g., whether the specific purpose and context of seeking information is highly relevant or less relevant for personal decisions). Thus, in the context of seeking information, cooperative behavior primarily refers to the management of different subtasks (e.g., searching with different keywords or in different databases) and leads to advantages regarding the division of labor. This form of cooperation among a group of information seekers should produce more and potentially more varied search results but would not necessarily promote any additional benefits beyond this.

In contrast, the benefits of collaboration in seeking information likely extend to another level, as experts in the field of collaboration in online learning posit that, in general, collaboration in online contexts can be considered both learning processes and outcomes (e.g., Oeberst et al. 2016). For instance, in the context of online information behavior, knowledge databases (such as Wikipedia) that were generated collaboratively by communities can be used by others; to these other users, it an outcome of the collaboration of former users. At the same time, these other users can also be a part of the collaborative process, which might lead to evolved knowledge databases (e.g., Détienne et al. 2016). Thus, here the collaboration is between information seekers who seek, evaluate, and use information collaboratively. Apparently, “collaboration is not a mere juxtaposition or collation of individual efforts” (Baker 2015, p. 5); instead, the processes within collaborative learning likely produce more than just the sum of each learner’s individual efforts and gains. Hence, from an educational point of view, *collaboration as a process* (and not merely cooperation) should play a pivotal role in successfully seeking online information, particularly via key communication activities during the phases of evaluating and using online information. In this sense, communication between information seekers might serve as means for seekers to more deeply elaborate the issues of interest, critically reflect on searching strategies and the sourcing of online information, and, finally, achieve higher quality learning outcomes.

## Learning through collaborative argumentation

A key educational activity that promotes outcomes like critical thinking (Guiller et al. 2008), problem-solving skills (Golanics and Nussbaum 2008), and learning about a particular topic (e.g., Andriessen 2006; Chinn and Clark 2013; Mason 2001) is *computer-mediated collaborative argumentation*. In these learning environments “learners communicate with each other via text-based […] discussion boards [and] are supposed to engage in argumentative discourses with the goal to acquire knowledge” (Weinberger and Fischer 2006, p. 71). Numerous studies confirm that dealing with complex content in collaborative argumentation can be conducive to learning in computer-mediated settings (Gerber et al. 2005; Muukkonen et al. 2005; Nussbaum et al. 2008; an overview is given in a meta-analysis by Noroozi et al. 2012). This positive effect depends on whether the experience offers certain conditions: the integration of multiple perspectives, transactivity in learners’ interaction, and the use of high-quality argumentation.

Regarding learners’ behavior in argumentative discourse, the *socio-cognitive approach* (based on Piaget’s theories) proposes that such situations of socio-cognitive conflict cause learners to solve these conflicts by engaging in reflection through cognitive reconstruction (*conceptual change*; see, e.g., Limón and Mason 2002; Sinatra and Pintrich 2003). This mechanism of deep knowledge elaboration causes learners to notice discrepancies in information or perspectives while socially interacting with others. This, in turn, allows learners to uncover misconceptions (Jucks and Paus 2013) and reconstruct their existing knowledge structures as well as establish new ones (Doise and Mugny 1984; von Aufschnaiter et al. 2008).

To successfully co-construct elaborated knowledge on a topic (e.g., Chi 2009; Teasley 1997), learners need to apply “reasoning that operates on the reasoning of another” (*transactive discourse*; Berkowitz and Gibbs 1983, p. 402). In particular, conflict-oriented consensus-building processes are described as being highly transactive (Fischer et al. 2002). The most important feature of this type of consensus building is that learners do not accept contributions of their partners as they are; instead, they engage in mutual discussion. Thus, the effectiveness of argumentative exchange critically depends on the discourse partners’ efforts to deeply elaborate on and challenge their partner’s knowledge and arguments (e.g., Kuhn and Udell 2003; in computer-mediated settings see Jucks and Paus 2013; Paus and Jucks 2012).

Learning can be promoted through verbal activities that involve interaction and co-construction, such as clarifying misunderstandings and or challenged by and challenging others (Asterhan and Schwarz 2007; Pena-Shaff and Nicholls 2004; Resnick et al. 2015; Paus et al. 2012). Drawing on Walton’s work (Walton 1989), argumentation strategies can be divided in two major categories – those relating to the construction and exposition of one’s own argument and those relating to the opponent’s position and claims (Felton and Kuhn 2001; Kuhn and Udell 2003). In the case of such counterarguments, the explicit and critical questioning of arguments and their associated evidence can enhance critical consumption of knowledge (Millar and Osborne 1998; Newton et al. 1999) as well as critical reflection on the quality of arguments (Watts et al. 1997; Nussbaum and Edwards 2011). Thus, critical questioning can be considered a strong argumentative strategy, given its capacity to address deeper grounds of disagreement and bring to light background knowledge and background beliefs that may otherwise escape attention.

In the context of seeking online information, the mechanisms of collaborative argumentation between information seekers might serve two purposes: 1) fostering information seekers’ conceptual understanding about the issue through mutual exchange and 2) critically questioning and discussing the sourcing of information and trustworthiness of information providers. Thus, the process of collaborative argumentation may offer many benefits for seeking online information and, thus, may help students develop a reflective and critical stance toward it.

## Technologies for collaborative argumentation when seeking online information

The process of collaborative information seeking can be greatly benefited by technology. First, tools can be deployed to support “offline” processes of information seeking (Leeder and Shah 2016), i.e., information seekers have opportunities to seek information within the same search task asynchronously or synchronously. In addition, to support cooperation and collaboration (see section 3 for the distinction between cooperation and collaboration) in seeking online information, technologies may also allow seekers to search, evaluate, and use online information collaboratively (e.g., Hertzum and Hansen 2019), in particular by allowing them to communicate with other information seekers. Communication may be supported using different kinds of technology, for instance instant messaging (e.g., Morris and Horvitz 2007), or chat systems (e.g., Mitsui et al. 2018).

According to Shah (2010), the notion of collaboration related to the specific area of seeking information consists of five components: communication, contribution, coordination, cooperation, and collaboration that can be supported by technology and may manifest in automatic components of the technology. While technologies for collaborative information seeking have not yet been systematically exploited, Jeong and Hmelo-Silver (2016) identified seven facets of computer-supported collaborative learning (CSCL) that can be supported by technology and that can expand the technological affordances described by Shah (2010) by addressing the general success of learning processes; these facets are (1) engagement of learners in a joint task, (2) communication, (3) resources contribution, (4) engagement of learners in productive collaborative learning processes, (5) engagement of learners in co-construction, (6) monitoring and regulation support for collaborative learning, and (7) group building.

Accordingly, the technology-enhanced support for CSCL and collaborative information seeking overlap (e.g., communication). As described above, communicative, contributive, coordinative, and cooperative processes are essential parts of collaborative processes, but each process per se does not necessarily involve or lead collaborative processes. Moreover, technological affordances, although they may allow users to perform certain learning processes more cooperatively (e.g., Le and Huse 2016) or even collaboratively, also do not necessarily lead users to do so (e.g., Jeong and Hmelo-Silver 2016). Thus, just because an automatic cooperation feature is integrated into a system does not mean that learners will necessarily learn cooperatively; it only means that the system allows for this to happen.

## Review of technologies for collaborative seeking online information

In order to make evidence-based and reasonable decisions, information seekers need to be supported in finding, selecting, and using online information (e.g., Ferrari 2013; Tabak 2015). Research on computer-supported collaborative learning emphasizes the potential of collaborative argumentation among learners (i.e., critical thinking/questioning, transactive dialogue, argumentation, integration of multiple perspectives) to help promote the successful seeking of online information as part of a learning process. As several technologies have emerged that aim to support the seeking of online information by allowing users to seek information collaboratively, the aim of this study is to review published articles about the use of these technologies in educational settings and to collate the above-described thoughts with how the articles considered (1) information seekers’ critical questioning of sourcing strategies when seeking online information, (2) their collaborative argumentation, and (3) the technological affordances available for seeking online information collaboratively. In this sense, we investigated research that focuses exclusively on processes of searching for online information as well research that focuses on the processes involved in seeking online information in the context of a broader learning process (where information seekers not only search for information but also find, evaluate, and use the information to learn).

- *RQ1 How do articles consider information seekers’ sourcing strategies when seeking online information?*
- *RQ2 How do articles consider collaborative argumentation among information seekers?*
- *RQ3 Which technological affordances are implemented to support seeking online information collaboratively?*

## Methods

### Sample

Our search was conducted in representative academic literature databases for psychology and pedagogy (e.g., Educational Resources Information Center 2004) in March 2019. We used EBSCOhost as provider for the research databases PsychARTICLES, Psychology and Behavioral Sciences Collection, PSYNDEX, PsychINFO, and Library Information Science and Technology Abstracts. Thus, by including research that was published in contexts of Library and Information Science, we extended our search, as we assumed research on seeking information collaboratively through technologies to be relevant in these fields, irrespective of any educational focus. To further detect research in educational contexts, we used ERIC, a predominant database on pedagogical research (Educational Resources Information Center 2004). In EBSCOhost, the option “publication types” was set to “Journals” and “Academic Journals” so that only peer-reviewed journal articles were included into our search. Similarly, we only searched for “Journal Articles” in ERIC.

The search keywords for all databases were: collaborat* information system AND collect* information search* OR collect* information seek* OR collect* information retriev* OR collect* information behavior OR collect* knowledge building OR collaborat* information search*OR collaborat* information seek* OR collaborat* information retriev* OR collaborat* information behavior OR collaborat* knowledge building. In EBSCOHOST, we added for ‘AND ‘Computer’ for collaborat* knowledge building’ to preliminarily filter articles without technologies. Similarly, in ERIC, we added the restriction AND ‘Computer’ for collaborat* information seek*, collaborat* information retriev*, collaborat* knowledge building to preliminarily filter articles without technologies. The reason for this additional restriction was that for these search terms, articles emerged that often use the term “system” as a sociological concept (e.g., to describe the classroom as a system).

In sum, our search according to these search terms resulted in 306 articles without duplicates. The initial search results for EBSCOhost included 218 articles, with most exact duplicates being removed from the results by EBSCOhost automatically and 4 exact duplicates being removed manually. From ERIC we obtained 88 articles, of which 52 were duplicated in ERIC and the EBSCOhost search. A first screening of these articles – including the article abstracts and the entire content of the articles – resulted in a limited sample of 102 articles. For this first screening, we used the following inclusion criteria: (1) The article describes the development or design of a technology being used for scientific purposes; (2) the article evaluated the technology scientifically; (3) the article made available all information about the deployed technology; (4) the technology was used exclusively to collaboratively solve information tasks or the technology was used to collaboratively solve information tasks as part of a complex learning task.

The 102 articles resulting from the first screening underwent a second screening, based again on their entire content. To the previous four criteria, we added the following criterium: The article’s main focus had to be on “collaboration as a process” rather than on “collaboration as an outcome”. The articles focusing on “collaboration as an outcome” (*n* = 7) investigated the use and development of knowledge bases that emerged through communities and, hence, were generated collaboratively (seekers use an outcome of collaboration), but in this review we focus on “collaboration as a process” (*n* = 87) and, therefore, on the processes between individuals that seek, evaluate, and use information collaboratively. Additionally, we included articles (*n* = 8) that focused on collaboration as both an outcome and a process. Thus, we reviewed *N* = 95 articles in terms of the research questions.

### Coding scheme

To investigate the research questions, we developed a coding scheme in line with the above-described approaches from pedagogical and psychological fields. The scheme included the category “study demographics” and further main categories of “collaboration”, “communication” and “technology” as well as “trust” to exploratorily investigate aspects specific to online information behavior.

#### Study demographics

The following categories were included under the dimension “study demographics” and were used to describe: (1) the *study design* of the article, meaning whether the design of the study was (quasi)experimental, descriptive, or naturalistic; (2) *search topic*, meaning what the field of the topic was, such as generic, academic research, socio-scientific issue, chemistry, or biology; (3) the *number of participants* if applicable, meaning how many participants worked together on the information task in the study; and (4) the *educational context*, meaning primary, middle, and secondary school, higher education, informal settings such as searches for personal information needs, or professional contexts such as medical professionals and teachers as educational professionals.

#### Consideration of information seekers’ sourcing strategies when seeking online information

The following categories were included under the dimension “trust” and were used to describe the articles’ *consideration of users’ sourcing strategies* in online information and the provider of online information, meaning whether the authors of an article or the participants referred to any strategies of sourcing online information and providers (e.g., assessment of credibility of information, or critical questioning of users’ use of information about the expertise of the source, the usability of media, or the ease by which the information could be understood (i.e., source information about the information)***.***

#### Consideration of collaborative argumentation

The following categories were included under the dimension “collaboration” and were used to describe whether the processes undertaken by information seekers (users or students, etc.) in each study were theoretically or methodologically considered *cooperation* (e.g., a division of labor), *collaboration* (e.g., co-constructive process among learners to gain knowledge), or a combination of both. In this context, an article was coded as focusing on collaboration if it referred to aspects of collaborative argumentation such as exchange of *multiple perspectives, argumentation, transactive dialogue,* or *(conflict-oriented) consensus building* in knowledge processes, or *critical thinking/questioning*.

The following categories were included under the dimension “communication” and were used to describe (1) the study’s *consideration of communication* theoretically or methodologically or as function in the technology, meaning whether the study did or did not consider communication between users and (2) whether the study considered the *contribution of communication* as a tool (e.g., tools to communicate are required to be integrated in the technologies) or as critical for investigating online information behavior processes (e.g., authors analyzed aspects of communication in order to investigate processes between information seekers). In this context, we further detected whether the article analyzed rather superficial aspects of communication (such as length of forum posts, or time spent on communication) or the content of communication by content analysis (such as how students answered their peers’ questions or whether critical questions were used more often after specific statements). See also Table 1 for explanations and examples of the categories.

**Table 1:** Coding scheme and examples

	Category	Explanation		Example	
Trust	Consideration of users’ trust	Article refers to trust or seekers’ reflection on sourcing strategies		“Social Q&A sites may have elements such as rating and recommender systems to help users judge one another’s trustworthiness or expertise, but they are not de- signed as formal expertise networks, where individuals have a way to identify potential collaborators, or people with similar information needs. “(p. 694) (Gazan, 2010)	
	No consideration of users’ trust	Article does not refer to trust or seekers’ reflection on sourcing strategies		–	
Collaboration	Collaboration	Article focus on aspects of collaboration, such as co-construction, integration of multiple perspectives, argumentation, critical questioning, or conflict-oriented consensus building		Defined as knowledge building level: “Using experience and literature to analyse and summarize. Negotiation of meaning or co-construction of knowledge “(p. 580) (Sun, Liu, Luo, Wu, & Shi, 2017)	
	Cooperation	Article focus on aspects of cooperation, such as a division of labor, or management of tasks		“analyzed the time duration of different behavior in each group, the relations between group members’ queries, the use of learning resources in different stages, as well as the labor division “(p. 3) (Wu, Liang, & Yu, 2018)	
Communication	No consideration of communication at all	Article does not consider communication, neither in theory, in technological function, nor in methods		–	
	Consideration of communication	As function in technology	Function is represented in tools to communicate integrated in the technologies	“[Coagmento] has bookmark, recommend, annotate, snip and editor functions, as well as a chat area, resource area, notepad and notification region.” (p. 234) (Wu, Liang, Dong, & Qiu, 2018)	
		In analysis	Rather superficial	Aspects such as length of forum posts, or time spend on communication	“Relations between group members’ queries. Every member of each group faced the same task, so the number of identical or similar queries reflects whether the group’s division of labor is reasonable and whether communication is timely to a certain degree. This could reflect how they searched for information in the learning process” (p. 8) (Wu, Liang, & Yu, 2018)
			Content-analysis	Aspects such as how students answered questions of their peers or whether critical questions were used more often after specific statements	“As explained in the previous section, textual contributions (chat contributions) are classified into five categories: (1) Suggestion (proposing components/properties, actions, ideas). (2) Evaluation (agreement, disagreement, other kind of feedback) of a previous action, suggestion, or evaluation from another learner. (3) Explanation by a learner of his/ her own actions. (4) Precision or clarification by a learner of his/her own suggestions or evaluations. (5) Question. (6) Answer” (p. 296) (Lonchcamp, 2009)

#### Technologies

The following categories were included under the dimension “technologies” and were used to describe the *specificity of the technologies*, meaning whether a technology was used exclusively for collaborative information seeking (e.g., Coagmento) or whether a technology was used in the process of learning, of which collaborative information seeking was a part (e.g., chats, wikis, forums, blogs), or whether a technology was used for a complex learning task that integrated processes of collaborative online information behavior (e.g., KnowledgeForum). By only focusing on technologies that were specific for collaboratively seeking online information, we included the following categories under the dimension *technology affordances for collaboration in seeking online information*: communication, contribution, coordination, cooperation, and collaboration (Shah 2009). Accordingly, the features of technologies were described regarding whether they allowed users to communicate, contribute, coordinate, cooperate, or collaborate.

### Analysis

Of the 95 articles, a second rater rated 24 articles (i.e., 25%) in terms of the main categories *consideration of information seekers’ sourcing strategies (trust)*, *collaboration*, and *communication* in order to determine the objectivity in the coding of articles (Campbell et al. 2013). The inter-rater reliability among both raters’ ratings ranges from Cohen’s kappa *k* = .859 to 1.00.

## Results

### Study demographics

In sum, 95 articles were analyzed, with (*N* = 70) articles retrieved on EBSCOhost. The publication dates of articles range from 1989 to 2019, where 51.6% of articles were published from 2013 on. While 53.7% of the articles (*N* = 51) followed a (quasi)experimental study design, a further 38.9% (*N* = 37) rather descriptively investigated the technologies without any manipulation, and a further 7.4% (*N* = 7) observed data in naturalistic settings. Moreover, 56.9% of the articles (*N* = 54) referred to the investigated “search topic” as generic, including three articles that investigated informal search tasks (such as everyday searches for travel information); conversely, 29.5% of the articles (*N* = 28) had “search topics” that related to academic research or education. In 7.4% of articles (*N* = 7), specific science topics such as chemistry were investigated, and 6.3% (*N* = 6) investigated more than one topic.

The numbers of participants investigated in the articles range from *N* = 0 to *N* = 1370 participants (*M* = 108.47; *SD* = 217.22), with 12 articles in which we were not able to identify the number of participants (e.g., because posts in online discussion boards were investigated). Lastly, 53.7% of the articles (*N* = 51) were conducted in higher education contexts, 16.9% were conducted in school settings (primary: *N* = 3; secondary: *N* = 7; and middle school: *N* = 6), 11.6% (*N* = 11) were conducted in professional contexts, and 11.6% (*N* = 11) were conducted in informal settings (4 articles not determinable).

### Descriptive results of the main categories

With respect how the articles considered seekers’ sourcing strategies for seeking online information, 92.6% of the articles (*N* = 88) did not mention seekers’ critical reflection about the trustworthiness of information, of the information provider, or related concepts (such as credibility and reliability). A mere *N* = 7 articles (7.4%) did mention these concepts, but only in the theory or discussion sections. Furthermore, none of the articles differentiated the stages of online information behavior and, thus, did not explicitly declare whether they were referring to the entire process of online information behavior or a specific part, such as searching, seeking, selecting, evaluating, integrating, or using the information.

With respect whether the articles considered collaborative argumentation, 21.1% of the articles (*N* = 88) focused only on aspects of cooperation, while a further 63.2% (*N* = 60) mainly focused on aspects of collaborative argumentation; 5.3% (*N* = 6) focused on aspects of both cooperation and collaboration (10 articles were undeterminable). In terms of communication, 16.8% of the articles (*N* = 16) did not consider communication at all, whereas 81.1% (*N* = 79) considered aspects of communication at least in theory, by analysis of communicative aspects, or as a function in the technology. Out of these 79 articles, 42.2% (*N* = 40) considered communication rather as a tool, as communication was only mentioned as a function in the technology, while 35.8% also analyzed aspects of communication to investigate how information seekers tended to communicate (analysis of superficial aspects: *N* = 25; content analysis: *N* = 9) (another 4 articles (4.2%) considered communication implicitly, but there was no communication function in the technology). In this sense, only 9 articles investigated aspects of collaborative argumentation within processes related to searching for information by analyzing the content of information seekers’ communication.

In terms of the specificity of the technologies, 34.7% of the articles (in sum *N* = 33; Coagmento: *N* = 11; others: 22) focused on systems that exclusively aim to support collaborative online information seeking, while 65.3% (in sum *N* = 62; KnowledgeForum: *N* = 15; others: 47) focused on a technology for a complex learning task that integrates the process of collaborative online information seeking. Out of all articles, 13.8% (*N* = 14) investigated a technological tool used in the process of collaborative information seeking (e.g., chats, wikis, forums, blogs).

### Relative frequencies and interdependence of the main categories

To investigate in more detail how specific aspects of collaborative argumentation, communication, and the technologies were considered in the articles, we investigated the articles’ focus on these items (collaborative argumentation, communication, and specificity of technologies) in terms of relative frequencies and interdependences.

A chi-square test was used to compare articles’ consideration of collaboration and specificity of technologies. No cell frequency was below 5. The minimum expected count was 6.25. Results show a significant difference in frequencies between articles’ consideration of collaboration and specificity of technologies, χ3(1) = 29.50, *p* < .001, φ < .607. While 51 out of the 55 articles that focused on systems that support general learning and integrate aspects of online information seeking also focused on aspects of collaborative argumentation, only 9 out of 25 articles that focused on systems that exclusively aim to support online information seeking focused on collaborative argumentation.

A second chi-square test was used to compare articles’ consideration of communication and specificity of technologies. One cell frequency was below 5. The minimum expected count was 3.13. Results show a significant difference in frequencies between articles’ consideration of communication and specificity of technologies, χ2(1) = 4.40, *p* = .036, φ < .234. While 51 out of the 55 articles focusing on technologies for a complex learning task that integrates the process of collaborative online information seeking considered communication among seekers either in theory, by analysis, or as function of the system, only 19 out of the 25 articles focusing on technologies that exclusively aim to support online information seeking considered any communication among the information seekers.

### Affordances of technologies that support collaborative seeking online information

Out of the reviewed technologies, eight single systems were identified to demonstrate tasks for collaborative seeking information (see Table 2), where “Y” indicates that a feature is supported by the technology, and the text in the brackets illustrates how affordances of technologies support a specific feature. Regarding *communication*, most technologies support communicative features by integrating a chat window (i.e., instant message function) or displaying comments by individual users in a window (e.g., AntWorld, SearchTogether). Two systems, PERCIRS and ARIADNE, do not support any communication feature. With respect to *contribution*, all technologies support users in sharing their search results (e.g., sharing URL, brief annotations). As to the *coordination* of collaborative information seeking technologies, we found that coordinative features are supported ranging from “loose” coordination mechanisms (e.g., using chat or instant message windows in the case of Searchbots, SearchTogether, and Coagmento, which simply permit users to coordinate search tasks) to more structured coordination mechanisms (i.e., coordination suggested by the technology in COLTIS and PERCIRS, which encourages users to coordinate search tasks). One of the advanced coordination approaches, exploited by the technology PERCIRS, calculated the similarity between user profiles.

**Table 2:** Affordances of technologies for seeking online information collaboratively

System	Communication	Contribution	Coordination	Cooperation	Collaboration
ARIADNE (Twidale et al., 1997)	–	Y (a series of command-output pairs)	–	–	–
AntWorld (Sun & Kantor, 2006)	Y (a relevance judgment)	Y (URLs of useful Web pages and brief annotations)	–	–	–
ColTIS (Mohammad Arif & Du, 2018)	Y (chat)	Y (query sharing, information sharing)	Y (division of search task)	Y (cooperative search)	Y (travel plan editing)
Coagmento (Mitsui et al. 2018)	Y (group chat)	Y (search results)	Y (chat)	–	–
PERCIRS (Naderi & Rumpler, 2010)	–	Y (relevant documents)	Y (automatic coordination by calculating the similarity between user profiles)	–	–
SearchX (Putra et al., 2018)	Y (chat)	Y (awareness components: bookmark sharing, page rating, annotation)	Y (division of labor)	–	Y (awareness & knowledge sharing components)
Searchbots (Avula et al., 2018)	Y (chat)	Y (chat)	Y (chat)	Y (give required information to the chatbot)	–
SearchTogether (Morris and Horvitz 2007)	Y (ratings and/or comments with a webpage, instant messaging)	Y (awareness components: per-user query histories, page-specific metadata)	Y (Instant messaging, recommendation queue)	Y (Plit search, multi-engine search)	Y (persistence, creating a shared artifact that summarizes the findings of a collaborative search)

Y = component(s) entailed in technologies

*Collaboration* is an important feature of a collaborative information seeking technologies, but not all technologies explicitly foster collaborative features (i.e., ARIADNE, AntWorld, and PERCIRS). ARIADNE and AntWorld were first developed for individual information seeking tasks and were then deployed in a collaborative setting. Thus, they do not support any collaborative features. PERCIRS does not support any collaborative features since it only has an automatic coordination component, and no communication between users is supported. Thus, a collaborative learning process among users (e.g., exchange of multiple perspectives) cannot take place and is rather coordinated superficially by the system. In contrast, the collaborative features of ColTIS, SearchX, and SearchTogether, for instance, allow users to edit common travel plans or create a shared artifact that summarizes the search findings – additionally, they allow users to communicate about the search results. In addition to investigating the five components of the collaborative information seeking process, we also investigated whether those technologies demonstrated educational impact. We found SearchTogether to be the only technology for collaborative information seeking that explicitly supports educational purposes; the aim of the system is that users can learn by performing collaborative information seeking tasks.

## Discussion

### Main findings

- *RQ1 How do articles consider information seekers’ sourcing strategies when seeking online information?*
- *RQ2 How do articles consider collaborative argumentation among information seekers?*
- *RQ3 Which technological affordances are implemented to support seeking online information collaboratively?*

Regarding RQ3, namely the specific technological affordances can support collaboration when seeking information, it seems that the feature of *contribution* (i.e., functions that allow users to share content) is obligatory for technologies that support collaborative information seeking, as all investigated technologies seem allow, for instance, the sharing of results. Looking at the features *cooperation and collaboration*, we found that only some technologies support both aspects (e.g., the task can be divided and negotiated between users). Likewise, and addressing RQ2, articles focusing on technologies that exclusively aim to support collaborative information seeking (i.e., not embedded in an entire learning process), considered fewer aspects of collaborative argumentation and fewer patterns of communication compared to articles focusing on technologies that considered information seeking as a part of a learning process. However, research on collaborative argumentation indicates that collaboration and cooperation models must be integrated into technologies that support collaborative information seeking, in the same way that these models are often considered in technologies that foster deeper learning and skill development (e.g., Loll and Pinkwart 2013).

Communicative behavior between learners, in itself, is not conducive to learning (Felton et al. 2015), but it becomes conducive to learning when it fulfils specific conditions, such as elaboration of the issue in question through collaborative argumentation. Accordingly, communication features for collaborative argumentation in information seeking need instructional and didactic support functions to motivate and promote learning (Mayweg-Paus et al. 2016; Thiebach et al. 2016; Vogel et al. 2017). This indicates that existing technologies aimed at supporting collaborative information seeking need to be researched and potentially extended with regard to their educational purposes.

In this sense, focusing particularly on the communication among seekers likely has the potential to promote successful seeking of online information, as fostering collaborative argumentation among learners (i.e., critical questioning, argumentation, integration of multiple perspectives, and transactive dialog) may not only help learners in searching for adequate information in order to learn but also in their learning about how to search adequately (e.g., with critical questioning one’s own sourcing strategies) (Rieh et al. 2016). For example, the more an information seeker understands the background of a topic, the better she might be able to search appropriately and to critically reflect on the sourcing of the information. Conversely, the more competent she is at reflecting on the sourcing of information, the deeper she should be able to elaborate the evidence of information. In this context, and related to RQ1, seekers’ reflection of sourcing strategies (i.e., trusting) seems to be underrepresented in the reviewed articles (Appendix Table 3).

### Limitations

The body of reviewed articles was limited to those found on pedagogical and educational databases, as we were particularly interested in technologies for collaborative information seeking that were investigated in educational settings. In this context, these investigations of single technologies might not cover information seekers’ actual use of technologies, as they often use a variety of tools, ranging from e-mail to video conferencing, to support their collaboration during information-seeking activities (e.g., Spence et al. 2005). For the investigation of these technologies’ affordances as related to collaborative information seeking, we classified technological components that support users’ communication, contribution, coordination, cooperation, or collaboration. By focusing on the technological components, we were not able to describe the way users use these affordances. For instance, the communication between users that use a chat function may be mainly characterized by guiding the division of search terms (i.e., cooperation), the sharing of single search results (i.e., contribution), or the exchanging of multiple perspectives on a search topic (i.e., collaboration). In this sense, a specific technological component (e.g., chat function) may not only foster user communication but may also help them engage in collaborative learning processes (e.g., by critical questioning the other seekers). Yet, a mere technological component that fosters user collaboration does not necessarily mean that users will use it to engage in collaborative learning processes. In order to describe the actual learning processes of users during collaborative information seeking – and, thus, also to be able to measure and promote specific aspects of learning processes – when applying single technological components, it seems fruitful to use theoretical frameworks to assume specific learning goals for collaborative and cooperative learning processes.

Furthermore, the focus in our investigation on whether the reviewed articles consider information seekers’ reflection of sourcing strategies does not represent the complexity of phenomena and research related to identifying and understanding online information seeking, as we did not address other aspects that are considered in research (e.g., psychological features of seekers, such as seekers’ attitudes: Nauroth et al. 2015; or seekers’ trust propensity: Lucassen and Schraagen 2012) (e.g., see Khosrowjerdi 2016; Marton and Choo 2012 for an overview of sum theories used in research on seeking online health information). In this context, other approaches that aim to highlight aspects of seeking information are highly relevant for online contexts (e.g., the fact that content can be filtered by algorithms, e.g., Shin and Park 2019; Zimmermann and Jucks 2018b).

### Implications

Taking into account research on computer-supported collaborative learning and information seekers’ critical reflection about sourcing strategies in online information, there is a crucial need for information seekers to critically question the sourcing of online information and to argue not only about the relevance of information but also about the sourcing of online information. Hence, technologies aimed at supporting collaborative information seeking would benefit immensely from adding functions that allow for mutual discussions (e.g., Siemon et al. 2019). Because users’ and designers’ understanding of the relevance of collaborative argumentation may increase the likelihood that they will communicate about the searching and learning processes (rather than just the information they find), future research should address whether information seekers who reflect on their searching strategies – including how they consider specific aspects of their online information seeking (e.g., awareness of algorithms-generated content, of the competence of people who provide information, or of the usability of media) – are more successful in their online information seeking. In this sense, future research could perform systematic investigations on whether collaborative argumentation can promote seekers’ reflections about searching strategies, seekers’ abilities to adequately search for information (learning to search), as well seekers’ abilities to acquire knowledge on a search topic (searching to learn).

Additionally, research needs to investigate how the embedded communication functions of technologies can be used by users in order to meaningfully communicate using collaborative argumentation. In order to develop and integrate support functions in any technology for collaborative online information seeking, developers should reference research on instructional support in computer-supported collaborative learning, as it represents a solid basis for deriving design elements. Here, studies on the integration of CSCL scripts (i.e., instructional support to guide learners by the use of technology about how to interact) provide interesting approaches for designing learning settings wherein learners perform meaningful and beneficial collaborative learning activities; for example, CSCL scripts that prompt *transactive activities* have been shown to have a positive significant effect on domain-specific knowledge acquisition (e.g., Vogel et al. 2017). In this sense, implementing prompts to a technology that guide users on how to engage with other leaners transactively may increase their co-constructional understanding.

In particular, educational scholars need to address both of the following processes in order to promote online information behavior in students: searching to learn, as an information- (or knowledge-)seeking activity that incorporates the process of argumentation, as well as learning to search, where argumentative dialogue works as a mechanism to help users critically reflect on their sourcing strategies, the information providers, and the media (e.g., von Aufschnaiter et al. 2008; Rieh et al. 2016).

In contrast to other related reviews of technologies that support collaborative online information seeking (Ghadirian and Salehi, 2018; Hertzum and Hansen 2019), this review investigates whether and how technologies are presented alongside the concept of collaborative argumentation among information seekers and how these technologies’ affordances can support collaborative information seeking. This review demonstrates that some reviewed technologies – but not all – use existing functions to allow for collaborative argumentation (e.g., chat windows, instant messaging). For these technologies, integrating functions that allow seekers to communicate – such functions that were mainly used in articles focusing on technologies for general learning processes – may also promote learning in the specific context of seeking online information.

Yet, these technologies might require additional instructional interventions as well as further technological support, such as prompting functions that can help users engage in critical reflection (Thiebach et al. 2016; Renner et al. 2016) and instructions on critical questioning (Mayweg-Paus et al. 2016; Heijltjes et al. 2015). For example, developers of such a technology could implement prompts that inform learners at certain steps of the learning process about background knowledge, including criteria that are important for collaborative argumentation. In this sense, in the beginning of a conversation, a technology could encourage users to increase each other’s understanding of perspectives (i.e., to increase common ground). Later during a conversation, a technology could instruct users to critically question each other’s arguments by, for instance, bringing contradictory evidence or by faulting the reasoning underlying the argument (e.g., Felton and Kuhn 2001).

Further, future research may also investigate whether coordination and collaboration in online information seeking can be supported by the use of pedagogical agents that, for example, play the role of a learning companion or a moderator between two actual learners, thus changing the dialogue policy from system-initiative to user-initiative (Jurafsky and Martin 2000). Here, scripts could be developed for these pedagogical agents that guide meaningful collaboration between users. Furthermore, because seeking online information (collaboratively) is often considered to be specific for different situations and for different features related to the information (e.g., within health sciences or economics and for information that differs in terms of content or task, complexity, ambiguity, and actuality or its relevance for information seekers) (e.g., Kellar et al. 2007; Zhao and Zhang 2017), future research could extend our understanding of the complexity of seeking online information collaboratively by also investigating whether specific aspects of collaborative argumentation produce different effects for different seeking situations.

Finally, considering an individual’s process of seeking online information to be exclusively independent of collaborative online information seeking processes likely ignores information seekers’ actual seeking behavior. In particular, research on seeking online information within social networks allows for assumptions about seekers’ active seeking strategies concerning how they seek information individually and collaboratively (e.g., Ramirez and Walther 2015; Zhao and Zhang 2017). Thus, this review’s integration of research on computer-supported collaborative learning and trust in individual information seeking may contribute to the understanding of general information seeking, as it may address transitions between the elements of seeking information individually and collaboratively (Hertzum 2017).

## Appendix

**Table 3:** Reviewed articles

No.	Titel	Author(s)	Journal	Publication	Doi
1	A three-level analysis of collaborative learning in dual-interaction spaces.	Lonchamp, Jacques	International Journal of Computer-Supported Collaborative Learning	2009	10.1007/s11412-009-9068-6
2	Activity theory as a framework for analysing knowledge building.	Van Aalst, Jan; Hill, Cher M.	Learning Environments Research	2006	10.1007/s10984-005-9000-6
3	An Exploratory Study of the Information-Seeking Activities of Adolescents in a Discussion Forum.	Gauducheau, Nadia	Journal of the Association for Information Science & Technology	2016	10.1002/asi.23359
4	Analyzing collaborative interactions: Divergence, shared understanding and construction of knowledge.	Puntambekar, Sadhana	Computers & Education	2006	10.1016/j.compedu.2004.10.012
5	Awareness in collaborative information seeking.	Shah, Chirag; Marchionini, Gary	Journal of the American Society for Information Science & Technology	2010	10.1002/asi.21379
6	Breakdowns in collaborative information seeking: A study of the medication process	Hertzum, Morten	Information Processing & Management	2010	10.1016/j.ipm.2009.10.005
7	Collaborative conceptualisation processes in the development of lightweight ontologies.	Sousa, Cristovao Dinis; Soares, Antonio Lucas; Pereira, Carla Sofia	VINE: The Journal of Information & Knowledge Management Systems	2016	10.1108/VJIKMS-03-2015-0022
8	Collaborative information searching as learning in academic group work.	Wu, Dan; Liang, Shaobo; Yu, Wenting	Aslib Journal of Information Management	2018	10.1108/AJIM-03-2017-0063
9	Collaborative information seeking in student group projects.	Leeder, Chris; Shah, Chirag	Aslib Journal of Information Management	2016	10.1108/AJIM-12-2015-0190
10	Collaborative knowledge building process: an activity theory analysis.	Singh, Gurparkash; Hawkins, Louise; Whymark, Greg	VINE: The Journal of Information & Knowledge	2009	10.1108/03055720911003987
11	Collaborative knowledge building with shared video representations.	Barthel, Ralph; Ainsworth, Shaaron; Sharples, Mike	International Journal of Human-Computer Studies	2013	10.1016/j.ijhcs.2012. 02.006
12	Collaborative knowledge building: A case study	Gilbert, Nancy J.; Driscoll, Marcy P.	Educational Technology Research and Development	2002	10.1007/BF02504961
13	Collaborative learning in wikis.	Chang, Yun-Ke; Morales-Arroyo, Miguel Angel; Than, Hla; Tun, Zarchi; Wang, Zhujun	Education for Information	2010	10.3233/EFI-2010-0910
14	Collaborative search in electronic health records.	Kai Zheng; Qiaozhu Mei; Hanauer, David A.	Journal of the American Medical Informatics Association	2011	10.1136/amiajnl-2011-000009
15	Cross-Evaluation: A new model for information system evaluation.	Ying Sun; Kantor, Paul B.	Journal of the American Society for Information Science & Technology	2006	10.1002/asi.20324
16	Design principles for distributed knowledge building processes.	Hewitt, Jim; Scardamalia, Marlene	Educational Psychology Review	1998	10.1023/A:1022810231840
19	Designing collaborative knowledge building environments accessible to all learners: Impacts and design challenges.	So, Hyo-Jeong; Seah, Lay Hoon; Toh-Heng, Hwee Leng	Computers & Education	2010	10.1016/j.compedu.2009.08.031
18	Developing metadiscourse through reflective assessment in knowledge building environments.	Lei, Chunlin; Chan, Carol K. K.	Computers & Education	2018	10.1016/j.compedu.2018.07.006
19	Effects of awareness on coordination in collaborative information seeking.	Shah, Chirag	Journal of the American Society for Information Science & Technology	2013	10.1002/asi.22819
20	Elementary students enhancing their understanding of energysaving through idea-centered collaborative knowledge-building scaffolds and activities.	Hong, Huang-Yao; Lin, Pei-Yi	Educational Technology Research & Development	2019	10.1007/s11423-9606-x
21	Engagement and knowledge building in an afterschool STEM Club: Analyzing youth and facilitator posting behavior on a social networking site.	Won, Samantha G.L.; Evans, Michael A.; Huang, Lixiao	Learning, Media and Technology	2017	10.1080/17439884.2015.1119161
22	Epistemic cooperation scripts in online learning environments: Fostering learning by reducing uncertainty in discourse?	Makitalo, Kati; Weinberger, Armin; Hakkinen, Paivi; Jarvela, Sanna; Fischer, Frank	Computers in Human Behavior	2005	10.1016/j.chb.2004.10.033
23	Evaluating collaborative information-seeking interfaces with a search-oriented inspection method and re-framed information seeking theory	Wilson, Max L.; schraefel, m.c.	Information Processing & Management	2010	10.1016/j.ipm.2009.10.001
24	Exploring the development of college students’ epistemic views during their knowledge building activities.	Hong, Huang-Yao; Chen, Bodong; Chai, Ching Sing	Computers & Education	2016	10.1016/j.compedu.2016.03.005
25	Factors affecting information seeking and evaluation in a distributed learning environment.	Lee, Jae-Shin; Cho, Hichang	Journal of Educational Technology & Society	2011	ERIC Number: EJ930246
26	Fostering a collaborative and creative climate in a college class through idea-centered knowledge-building.	Hong, Huang-Yao; Chang, Yu-Hui; Chai, Chin Sing	Instructional Science	2014	10.1007/s11251-013-9289-y
27	Fostering collective and individual learning through knowledge building.	Zhao, Ke; Chan, Carol K. K.	International Journal of Computer-Supported Collaborative Learning	2014	10.1007/s11412-013-9188-x
28	Harnessing collective knowledge inherent in tag clouds.	Cress, U.; Held, C.	Journal of Computer Assisted Learning	2013	10.1111/j.1365-2729.2012.00491.x
29	How collaborators make sense of tasks together: A comparative analysis of collaborative sensemaking behavior in collaborative information-seeking tasks.	Tao, Yihan; Tombros, Anastasios	Journal of the Association for Information Science & Technology	2017	10.1002/asi.23693
30	How scholars use academic social networking services.	Bardakcı, Salih; Arslan, Omer; Unver, Tuğba Kocadağ	Information Development	2018	10.1177/0266666917712108
31	Impact of Task Types on Collaborative Information Seeking Behavior.	Wu, Dan; Liang, Shaobo; Dong, Jing; Qiu, Jin	Libri: International Journal of Libraries & Information Services	2018	10.1515/libri-2017-0051
32	Individual learning and collaborative knowledge building with shared digital artifacts (PSYNDEXshort)	Kimmerle, Joachim; Moskaliuk, Johannes; Cress, Ulrike	International Journal of Behavioral, Cognitive, Educational and Psychological Sciences	2008	10.5281/zenodo.1333921
33	Influence of participation, facilitator styles, and metacognitive reflection on knowledge building in online university courses.	Cacciamani, Stefano; Cesareni, Donatella; Martini, Francesca; Ferrini, Tiziana; Fujita, Nobuko	Computers & Education	2012	10.1016/j.compedu.2011.10.019
34	Investigating impacts of spatial configurations on collaborative writing.	Shah, Craig; Gonzalez-Ibanez, Roberto; Read, Pam	First Monday	2015	10.5210/fm.v20i12.5872
35	Knowledge building and the quantity, content and quality of the interaction and participation of students in an online collaborative learning environment.	Yucel, Ummuhan Avcı; Usluel, Yasemin Kocak	Computers & Education	2016	10.1016/j.compedu.2016.02.015
36	Knowledge building and vocabulary growth over two years, Grades 3 and 4.	Sun, Yanqing; Zhang, Jianwei; Scardamalia, Marlene	Instructional Science	2008	10.1007/s11251-008-9082-5
37	Learning chemistry through the use of a representation-based knowledge building environment.	Schank, Patricia; Kozma, Robert	Journal of Computers in Mathematics and Science Teaching	2002	https://www.learntechlib.org/primary/p/9262/.
38	Learning to monitor and regulate collective thinking processes.	Borge, Marcela; Ong, Yann Shiou; Rose, Carolyn Penstein	International Journal of Computer-Supported Collaborative Learning	2018	10.1007/s11412-018-9270-5
39	Microcollaborations in a social Q&A community	Gazan, Rich	Information Processing & Management	2010	10.1016/j.ipm.2009.10.007
40	MineRank: Leveraging users? latent roles for unsupervised collaborative information retrieval.	Soulier, Laure; Tamine, Lynda; Shah, Chirag	Information Processing & Management	2016	10.1016/j.ipm.2016.05.002
41	PERCIRS: a system to combine personalized and collaborative information retrieval.	Naderi, Hassan; Rumpler, Beatrice	Journal of Documentation	2010	10.1108/00220411011052948
42	Reading for idea advancement in a Grade 4 knowledge building community.	Zhang, Jianwei; Sun, Yanqing	Instructional Science	2011	10.1007/s11251-010-9135-4
43	Relationships among tasks, collaborative inquiry processes, inquiry resolutions, and knowledge outcomes in adolescents during guided discovery-based game design in school.	Reynolds, Rebecca B.	Journal of Information Science	2016	10.1177/0165551515614537
44	Role taking and knowledge building in a blended university course.	Cesareni, Donatella; Cacciamani, Stefano; Fujita, Nobuko	International Journal of Computer-Supported Collaborative Learning	2016	10.1007/s11412-015-9224-0
45	Scaffolding wiki?supported collaborative learning for small?group projects and whole?class collaborative knowledge building.	Lin, C-Y.; Reigeluth, C. M.	Journal of Computer Assisted Learning	2016	10.1111/jcal.12140
46	Search on surfaces: Exploring the potential of interactive tabletops for collaborative search tasks	Morris, Meredith Ringel; Fisher, Danyel; Wigdor, Daniel	Information Processing & Management	2010	10.1016/j.ipm.2009.10.004
47	Sensemaking in Collaborative Web Search.	Paul, SharodaA.; Morris, MeredithRingel	Human-Computer Interaction	2011	10.1080/07370024.2011.559410
48	Social Justice as Strategy: Connecting School Libraries, Collaboration, and IT.	Dadlani, Punit; Todd, Ross J.	Library Quarterly	2016	10.1086/684143
49	Social question answering: Analyzing knowledge, cognitive processes and social dimensions of micro-collaborations.	Blooma, Mohan John; Kurian, Jayan Chirayath; Chua, Alton Yeow Kuan; Goh, Dion Hoe Lian; Lien, Nguyen Huong	Computers & Education	2013	10.1016/j.compedu.2013.07.006
50	Socially augmented argumentation tools: Rationale, design and evaluation of a debate dashboard.	Iandoli, Luca; Quinto, Ivana; De Liddo, Anna; Shum, Simon Buckingham	International Journal of Human-Computer Studies	2014	10.1016/j.ijhcs.2013. 08.006
51	Strategies, obstacles, and attitudes: student collaboration in information seeking and synthesis projects.	Leeder, Chris; Shah, Chirag	Information Research	2016	ERIC Number: EJ1114155
52	Student-directed assessment of knowledge building using electronic portfolios.	van Aalst, Jan; Chan, Carol K. K.	Journal of the Learning Sciences	2007	10.1080/10508400701193697
53	Students’ views of collaboration and online participation in knowledge forum.	Chan, Carol K. K.; Chan, Yuen-Yan	Computers & Education	2011	10.1016/j.compedu.2010.09.003
54	Sustaining knowledge building as a principle-based innovation at an elementary school.	Zhang, Jianwei; Hong, Huang-Yao; Scardamalia, Marlene; Teo, Chew Lee; Morley, Elizabeth A.	Journal of the Learning Sciences	2011	10.1080/10508406.2011.528317
55	Teacher collaboration network in Higher Education: Reflective visions from praxis.	Romeu, Teresa; Guitert, Montse; Sangra, Albert	Innovations in Education and Teaching International	2016	10.1080/14703297.2015.1025807
56	Testing an integrative theoretical model of knowledge-sharing behavior in the context of Wikipedia.	Hichang Cho; MeiHui Chen; Siyoung Chung	Journal of the American Society for Information Science & Technology	2010	10.1002/asi.21316
57	The dynamics of an online knowledge building community: A 5-year longitudinal study.	Myllari, Jarkko; Ahlberg, Mauri; Dillon, Patrick	British Journal of Educational Technology	2010	10.1111/j.1467-8535.2009.00972.x
58	The dynamics of team cognition: A process-oriented theory of knowledge emergence in teams.	Grand, James A.; Braun, Michael T.; Kuljanin, Goran; Kozlowski, Steve W. J.; Chao, Georgia T.	Journal of Applied Psychology	2016	10.1037/apl0000136
59	The Effects of Representation Tool (Visible-Annotation) types to support knowledge building in computer-supported	Shin, Yoonhee; Kim, Dongsik; Jung, Jaewon	Journal of Educational Technology & Society	2018	http://www.jstor.org/stable/26388383
60	The Impacts of Mutual Collaboration Experience and Domain Knowledge Levels on CAIS Behavior: An Experimental Study.	Dan Wu; Shaobo Liang; Xue Xiang	Libri: International Journal of Libraries & Information Services	2017	10.1515/libri-2016-0061
61	The orchestration of a collaborative information seeking learning task.	Knight, Simon; Rienties, Bart; Littleton, Karen; Tempelaar, Dirk; Mitsui, Matthew; Shah, Chirag	Information Retrieval Journal	2017	10.1007/s10791-017-9304-z
62	The role of e?portfolios in supporting productive learning.	Yang, Min; Tai, Mui; Lim, Cher Ping	British Journal of Educational Technology	2016	10.1111/bjet.12316
63	The role of scaffolding in CSCL in general and in specific environments.	Verdu, N.; Sanuy, J.	Journal of Computer Assisted Learning	2014	10.1111/jcal.12047
64	The role of team cognition in collaborative information seeking.	McNeese, Nathan J.; Reddy, Madhu C.	Journal of the Association for Information Science & Technology	2017	10.1002/asi.23614
65	Towards a probabilistic model for supporting collaborative information access.	Bohm, Thilo; Klas, Claus-Peter; Hemmje, Matthias	Information Retrieval Journal	2016	10.1007/s10791-016-9285-3
66	Tracing knowledge co-evolution in a realistic course setting: A wiki-based field experiment.	Kump, Barbara; Moskaliuk, Johannes; Dennerlein, Sebastian; Ley, Tobias	Computers & Education	2013	10.1016/j.compedu.2013.06.015
67	Understanding collaborative learning processes in new learning environments.	Hmelo-Silver, Cindy E.; Chernobilsky, Ellina; Jordan, Rebecca	Instructional Science	2008	10.1007/s11251-008-9063-8
68	Understanding tourists’ collaborative information retrieval behavior to inform design.	Mohammad Arif, Abu Shamim; Du, Jia Tina; Lee, Ivan	Journal of the Association for Information Science & Technology	2015	10.1002/asi.23319
69	Visualizing co-evolution of individual and collective knowledge.	Kimmerle, Joachim; Moskaliuk, Johannes; Harrer, Andreas; Cress, Ulrike	Information, Communication & Society	2010	10.1080/13691180903521547
70	When two heads are better than one.	Na, Kyoungsik; Lee, Jisu	Aslib Journal of Information Management	2016	10.1108/AJIM-04-2015-0057
71	The Collective Knowledge of Social Tags: Direct and Indirect Influences on Navigation, Learning, and Information Processing	Cress, Ulrike; Held, Christoph; Kimmerle, Joachim	Computers & Education	2013	10.1016/j.compedu.2012.06.015
72	Constructive Disruptions for Effective Collaborative Learning: Navigating the Affordances of Social Media for Meaningful Engagement	Rambe, Patient	Electronic Journal of e-Learning	2012	ERIC Number: EJ969451
73	A Theoretical Framework for Building Online Communities of Practice with Social Networking Tools	Gunawardena, Charlotte N.; Hermans, Mary Beth; Sanchez, Damien; Richmond, Carol; Bohley, Maribeth; Tuttle, Rebekah	Educational Media International	2009	10.1080/09523980802588626
74	Forming Shared Inquiry Structures to Support Knowledge Building in a Grade 5 Community	Tao, Dan; Zhang Jianwei	Instructional Science	41,817	10.1007/s11251-018-9462-4
75	Teachers’ Pedagogical Designs for Technology-Supported Collective Inquiry: A National Case Study	Lakkala, M; Lallimo, J; Hakkarainen, K	Computers and Education	2005	10.1016/j.compedu.2005.04.010
76	Semantic Search of Tools for Collaborative Learning with the Ontoolsearch System	Vega-Gorgojo, Guillermo; Bote-Lorenzo, Miguel L; Asensio-Perez, Juan I; Gomez-Sanchez, Eduardo; Dimitriadis, Yannis A; et	Computers & Education	2010	10.1016/j.compedu.2009.09.012
77	Finding and Reusing Learning Materials with Multimedia Similarity Search and Social Networks	Little, Suzanne; Ferguson, Rebecca; Ruger, Stefan	Technology, Pedagogy and Education	2012	doi.org/10.1080/1475939X.2012.698378
78	Study of the Impact of Collaboration among Teachers in a Collaborative Authoring System	Lafifi, Yacine; Touil, Ghassen	Journal of Information Technology Education	2010	ERIC Number: EJ895863
79	Limitless Learning: Assessing Social Media Use for Global Workplace Learning	Breunig, Karl Joachim	Learning Organization	2016	10.1108/TLO-07-2014-0041
80	Browsing Is a Collaborative Process	Twidale, M. B.; Nichols, D. M.; Paice, C. D.	Information Processing & Management	1997	10.1016/S0306-4573(97)00040-X
81	Modelling Situated Actions in Collaborative Hypertext Databases	Chen, Chaomei; Rada, Roy	Journal of Computer-Mediated Communication	1996	10.1111/j.1083-6101.1996.tb00189.x
82	Understanding Student Language: An Unsupervised Dialogue Act Classification Approach	Ezen-Can, Aysu; Boyer, Kristy Elizabeth	Journal of Educational Data Mining	2015	https://jedm.educationaldatamining.org/index.php/JEDM/article/view/JEDM095
83	Dancing with the Web: Students Bring Meaning to the Semantic Web	Brooks, Pauline	Technology, Pedagogy & Education	2012	10.1080/1475939X.2012.697400
84	Ontology Enabled Annotation and Knowledge Management for Collaborative Learning in Virtual Learning Community	Yang, Stephen J. H.; Chen, Irene Ya-Ling; Shao, Norman W. Y.	J Educ Techno Soc (Journal of Educational Technology & Society)	2004	ERIC Number: EJ853746
85	Wearable Learning Tools	Bowskill, Jerry; Dyer, Nick	ALT-J: Research in Learning Technology	1999	/10.1080/0968776990070305
86	Retrieval Hierarchies in Hypertext	Rada, R.; Sang, W.; Birchall, A.	Information Processing & Management	1993	10.1016/0306-4573(93)90061-H
87	Supporting Collaboration in Hypermedia: Issues and Experiences	Irish, Peggy M.; Trigg, Randall H.	Journal of the American Society for Information Science	1989	https://doi.org/10.1002/(SICI)1097-4571(198905)40:3 < 192::AIDASI10 > 3.0.CO;2–5
88	Exploring Collaborative Learning Effect in Blended Learning Environments	Sun, Z.; Liu, R.; Luo, L.; Wu, M.; Shi, C.	Journal of Computer Assisted Learning	2017	10.1111/jcal.12201
89	Enhancing Collaborative Learning in Web 2.0-Based ELearning Systems: A Design Framework for Building Collaborative E-Learning Contents	El Mhouti, Abderrahim; Nasseh, Azeddine; Erradi, Mohamed; Vasquez, Jose Marfa	Education and Information Technologies	2016	10.1007/s10639-016-9545-2
90	Collaborative Knowledge Building with Wikis: The Impact of Redundancy and Polarity	Moskaliuk, Johannes; Kimmerle, Joachim; Cress, Ulrike	Comput Educ (Computers & Education)	2012	10.1016/j.compedu.2011.11.024
91	Designing and Building a Collaborative Library Intranet for All	Battles, Jason J.	Journal of Web Librarianship	2010	10.1080/19322909.2010.500570
92	Incidence of group awareness information on students? collaborative learning processes	Pifarre, Manoli; Cobos, Ruth; Argelagos, Esther	Journal of Computer Assisted Learning	2014	10.1111/jcal.12043
93	Facilitating Social Collaboration in Mobile Cloud-Based Learning: A Teamwork as a Service (TaaS) Approach	Sun, Geng; Shen, Jun	IEEE Transactions on Learning Technologies	2014	10.1109/TLT.2014.2340402
94	Using blogs to support learning during internship	Chu, Samuel K. W.; Chan, Carol K. K.; Tiwari, Agnes F. Y.	Comput Educ (Computers & Education)	2012	10.1016/j.compedu.2011.08.027
95	Fostering student nurses? Self-regulated Learning with the Second Life Environment: An empirical study	Al-Samarraie, Hosam; Al-hatem, Ahmed; Masood, Mona	Journal of Information Technology Education: Research	2018	10.28945/Tabek4110

## References

[CR1] Aljazzaf, Z. M., Perry, M., & Capretz, M. A. M. (2010). Online trust: Definition and principles. Paper presented at the 5^th^ international multi-conference on computing in the global information technology, Valencia, Spain. 10.1109/ICCGI.2010.17.

[CR2] Alterman R, Harsch K (2017). A more reflective form of joint problem solving. International Journal of Computer-Supported Collaborative Learning.

[CR3] Andriessen J, Sawyer RK (2006). Arguing to learn. The Cambridge handbook of the learning sciences.

[CR4] Asterhan CSC, Schwarz BB (2007). The effects of monological and dialogical argumentation on concept learning in evolutionary theory. Journal of Educational Psychology.

[CR5] Avula, S., Chadwick, G., Arguello, J., & Capra, R. (2018). SearchBots: user engagement with chatBots during collaborative search. 10.1145/3176349.3176380

[CR6] Baker MJ (2015). Collaboration in collaborative learning. Interaction Studies: Social Behaviour and Communication in Biological and Artificial Systems.

[CR7] Berkowitz MW, Gibbs JC (1983). Measuring the developmental features of moral discussion. Merrill-Palmer Quarterly.

[CR8] Brante EW (2019). A multiple-case study on students’ sourcing activities in a group task. Cogent Education.

[CR9] Bråten I, Brante EW, Strømsø HI (2019). Teaching sourcing in upper secondary school: A comprehensive sourcing intervention with follow-up data. Reading Research Quarterly.

[CR10] Bromme R, Goldman SR (2014). The public’s bounded understanding of science. Educational Psychologist.

[CR11] Cacioppo JT, Petty RE, Kao CF, Rodriguez R (1986). Central and peripheral routes to persuasion: An individual difference perspective. Journal of Personality and Social Psychology.

[CR12] Caena F, Redecker C (2019). Aligning teacher competence frameworks to 21st century challenges: The case for the European digital competence framework for educators (Digcompedu). European Journal of Education.

[CR13] Campbell J, Osserman J, Pedersen O (2013). Coding in-depth semi-structured interviews: Problems of unitization and intercoder reliability and agreement. Sociological Methods and Research.

[CR14] Cash T, Desbrow B, Leveritt M, Ball L (2014). Utilization and preference of nutrition information sources in Australia. Health Expectations.

[CR15] Chaiken S (1980). Heuristic versus systematic information processing and the use of source versus message cues in persuasion. Journal of Personality and Social Psychology.

[CR16] Chen, J., Wang, M., Kirschner, P. A., & Tsai, C. C. (2018). The role of collaboration, computer use, learning environments, and supporting strategies in CSCL: A meta-analysis. *Review of Educational Research*, 799–843. 10.3102/0034654318791584.

[CR17] Chen YJ, Chien HM, Kao CP (2019). Online searching behaviours of preschool teachers: A comparison of pre-service and in-service teachers’ evaluation standards and searching strategies. Asia-Pacific Journal of Teacher Education.

[CR18] Chi MTH (2009). Active-constructive-interactive: A conceptual framework for differentiating learning activities. Topics in Cognitive Science.

[CR19] Chinn CA, Clark DB, Hmelo-Silver CE, Chinn CA, Chan CKK, O’Donnell AM (2013). Learning through collaborative argumentation. The international handbook of collaborative learning.

[CR20] Choi W, Stvilia B (2015). Web credibility assessment: Conceptualization, operationalization, variability, and models. Journal of the Association for Information Science and Technology.

[CR21] Détienne F, Baker M, Fréard D, Barcellini F, Denis A, Quignard M (2016). The descent of Pluto: Interactive dynamics, specialisation and reciprocity of roles in a Wikipedia debate. International Journal of Human Computer Studies.

[CR22] Doise W, Mugny G (1984). The social development of the intellect.

[CR23] Educational Resources Information Center, & EBSCO Publishing (2004). ERIC (Online).

[CR24] Eysenbach G, Köhler C (2002). How do consumers search for and appraise health information on the world wide web? Qualitative study using focus groups, usability tests, and in-depth interviews. British Medical Journal.

[CR25] Farooq U, Ganoe CH, Carroll JM, Giles CL (2009). Designing for e-science: Requirements gathering for collaboration in CiteSeer. International Journal of Human-Computer Studies.

[CR26] Felton M, Kuhn D (2001). The development of argumentive discourse skill. Discourse Processes.

[CR27] Felton M, Crowell A, Liu T (2015). Arguing to agree: Mitigating my-side bias through consensus-seeking dialogue. Written Communication.

[CR28] Ferrari, A. (2013). DIGICOMP: A framework for developing and understanding digital competence in Europe. Luxembourg: JRC Scientific and Policy Reports EUR26036EN.

[CR29] Fischer F, Bruhn J, Gräsel C, Mandl H (2002). Fostering collaborative knowledge construction with visualization tools. Learning and Instruction.

[CR30] Forte A (2015). The new information literate: Open collaboration and information production in schools. International Journal of Computer-Supported Collaborative Learning.

[CR31] Gerber S, Scott L, Clements DH, Sarama J (2005). Instructor influence on reasoned argument in discussion boards. Educational Technology Research and Development.

[CR32] Ghadirian, H., Salehi, K., & Ayub, A. F. M. (2018). Social annotation tools in higher education: A preliminary systematic review. *International Journal of Learning Technology, 13*(2), 130–162. 10.1504/IJLT.2018.092096

[CR33] Golanics JD, Nussbaum EM (2008). Enhancing online collaborative argumentation through question elaboration and goal instructions. Journal of Computer Assisted Learning.

[CR34] Gonzalez-Teruel A, González-Alcaide G, Barrios M, Abad-García MF (2015). Mapping recent information behavior research: An analysis of co-authorship and co-citation networks. Scientometrics.

[CR35] Griffin RJ, Dunwoody S, Neuwirth K (1999). Proposed model of the relationship of risk information seeking and processing to the development of preventive behaviors. Environmental Research.

[CR36] Guiller J, Durndell A, Ross A (2008). Peer interaction and critical thinking: Face-to-face or online discussion?. Learning and Instruction.

[CR37] Hargittai, E., Fullerton, L., Menchen-Trevino, E., & Thomas, K. Y. (2010). Trust online: Young adults' evaluation of web content. *International Journal of Communication, 4*, 468–494. Retrieved from: http://ijoc.org/index.php/ijoc/article/view/636/423 (WebCite: http://www.webcitation.org/6xWGL9e4T).

[CR38] Hattie JAC (2009). Visible learning: A synthesis of over 800 meta-analyses relating to achievement.

[CR39] Heijltjes A, van Gog T, Leppink J, Paas F (2015). Unraveling the effects of critical thinking instructions, practice, and self-explanation on students’ reasoning performance. Instructional Science.

[CR40] Hertzum M (2017). Collaborative information seeking and expertise seeking: Different discourses about similar issues. Journal of Documentation.

[CR41] Hertzum M, Hansen P (2019). Empirical studies of collaborative information seeking: A review of methodological issues. Journal of Documentation.

[CR42] Hilligoss, B., & Rieh, S. Y. (2008). Developing a unifying framework of credibility assessment: Construct, heuristics, and interaction in context. Information Processing and Management, 44(4), 1467–1484. 10.1016/j.ipm.2007.10.001.

[CR43] Iding MK, Crosby ME, Auernheimer B, Barbara Klemm E (2008). Web site credibility: Why do people believe what they believe?. Instructional Science.

[CR44] Jeong H, Hmelo-Silver CE (2016). Seven affordances of computer-supported collaborative learning: How to support collaborative learning? How can technologies help?. Educational Psychologist.

[CR45] Jones HS, Moncur W, McAlaney J, Frumkin L, Benson V (2018). The role of psychology in understanding online trust. Psychological and behavioral Examinations in Cyber Security.

[CR46] Jucks R, Paus E (2013). Different words for the same concept: Learning collaboratively from multiple documents. Cognition and Instruction.

[CR47] Jurafsky, D., & Martin, J. H. (2000). *Speech and language processing: An introduction to natural language processing, computational linguistics, and speech recognition* (1st ed.). Prentice Hall PTR.

[CR48] Kellar M, Watters C, Shepherd M (2007). A field study characterizing web-based information-seeking tasks. Journal of the American Society for Inforamtion Science and Technology.

[CR49] Khosrowjerdi M (2016). A review of theory-driven models of trust in the online health context. IFLA Journal.

[CR50] Kuhlthau CC (1993). Seeking meaning: A process approach to library and information services.

[CR51] Kuhn D, Udell W (2003). The development of argument skills. Child Development.

[CR52] Kyndt E, Raes E, Lismont B, Timmers F, Cascallar E, Dochy F (2013). A meta-analysis of the effects of face-to-face cooperative learning. Do recent studies falsify or verify earlier findings?. Educational Research Review.

[CR53] Le, N. T. & Huse, N. (2016). Evaluation of the formal models for the Socratic method. In *Proceedings of the 13th Conference on Intelligent Tutoring Systems*, Springer Verlag, pp 69–78.

[CR54] Leeder C, Shah C (2016). Collaborative information seeking in student group projects. Aslib Journal of Information Management.

[CR55] Limón M, Mason L (2002). *Reconsidering conceptual change: Issues in theory and practice* (pp. 115–135).

[CR56] List A, Alexander PA (2017). Cognitive affective engagement model of multiple source use. Educational Psychologist.

[CR57] Loll F, Pinkwart N (2013). LASAD: Flexible representations for computer-based collaborative argumentation. International Journal of Human Computer Studies.

[CR58] Lucassen T, Schraagen JM (2012). Propensity to trust and the influence of source and medium cues in credibility evaluation. Journal of Information Science.

[CR59] Marton C, Choo CW (2012). A review of theoretical models of health information seeking on the web. Journal of Documentation.

[CR60] Mason L (2001). Introducing talk and writing for conceptual change: A classroom study. Learning and Instruction.

[CR61] Mayweg-Paus E, Thiebach M, Jucks R (2016). Let me critically question this! – Insights from a training study on the role of questioning on argumentative discourse. International Journal of Educational Research.

[CR62] Metzger MJ, Flanagin AJ (2013). Credibility and trust of information in online environments: The use of cognitive heuristics. Journal of Pragmatics.

[CR63] Metzger MJ, Flanagin AJ, Medders RB (2010). Social and heuristic approaches to credibility evaluation online. Journal of Communication.

[CR64] Millar R, Osborne JF (1998). Beyond 2000: Science education for the future.

[CR65] Mitsui, M., Liu, J., & Shah, C. (2018). Coagmento: Past, Present, and Future of an Individual and Collaborative Information Seeking Platform. In CHIIR ‘18: 2018 Conference on Human Information Interaction Retrieval, March 11–15, 2018, New Brunswick, NJ, USA. ACM, New York, NY, USA, Article 4. 10.1145/3176349.3176896.

[CR66] Mohammad Arif, A. S., & Du, J. T., & Lee, I. (2018). Understanding Tourists’ Collaborative Information Retrieval Behavior to Inform Design. *Journal of the Association for Information Science and Technology, 66*(11), 2285–2303. 10.1002/asi.23319

[CR67] Morris MR, Horvitz E (2007). SearchTogether: An interface for collaborative web search. Proceedings of the 20th annual ACM symposium on user interface software and technology (UIST '07).

[CR68] Muukkonen H, Lakkala M, Hakkarainen K (2005). Technology-mediation and tutoring. How do they shape progressive inquiry discourse?. The Journal of the Learning Sciences.

[CR69] Naderi, H. and Rumpler, B. (2010), “PERCIRS: a system to combine personalized and collaborative information retrieval”. *Journal of Documentation, 66*(4), 532–562. 10.1108/00220411011052948

[CR70] Nauroth P, Gallwitzer M, Bender J, Rothmund T (2015). Social identity threat motivates science-discrediting online comments. PLoS One.

[CR71] Newton P, Driver R, Osborne J (1999). The place of argumentation in the pedagogy of school science. International Journal of Science Education.

[CR72] Noroozi O, Weinberger A, Biemans HJ, Mulder M, Chizari M (2012). Argumentation-based computer supported collaborative learning (ABCSCL). A synthesis of 15 years of research. Educational Research Review.

[CR73] Nussbaum EM, Edwards OV (2011). Critical questions and argument stratagems: A framework for enhancing and *analyzing students' reasoning practices*. Journal of the Learning Sciences.

[CR74] Nussbaum EM, Sinatra GM, Poliquin A (2008). Role of epistemic beliefs and scientific argumentation in science learning. International Journal of Science Education.

[CR75] Nygren, T., & Guath, M. (2019). *Swedish teenagers’ difficulties and abilities to determine digital news credibility*. *40*(2019), 23–42. 10.2478/nor-2019-0002.23.

[CR76] Oeberst A, Cress U, Back M, Nestler S, Cress U, Jeong H, Moskaliuk J (2016). Individual vs. collaborative information processing: The case of biases in Wikipedia. Mass collaboration and education.

[CR77] Paus E, Jucks R (2012). Common ground? How the encoding of specialist vocabulary impacts on peer-to-peer online discourse. Discourse Processes.

[CR78] Paus E, Werner CS, Jucks R (2012). Learning through online peer discourse: Structural equation modeling points to the role of discourse activities in individual understanding. Computers & Education.

[CR79] Pena-Shaff JB, Nicholls C (2004). Analyzing student interactions and meaning construction in computer bulletin board discussions. Computers & Education.

[CR80] Pérez A, Potocki A, Stadtler M, Macedo-Rouet M, Paul J, Salmerón L, Rouet JF (2018). Fostering teenagers’ assessment of information reliability: Effects of a classroom intervention focused on critical source dimensions. Learning and Instruction.

[CR81] Pian W, Khoo CS, Chang YK (2016). The criteria people use in relevance decisions on health information: An analysis of user eye movements when browsing a health discussion forum. Journal for Medical Internet Research.

[CR82] Purdyet E, Thoma B, Bednarczyk J, Migneault D, Sherbino J (2015). The use of free online educational resources by Canadian emergency medicine residents and program directors. Canadian Journal of Emergency Medicine.

[CR83] Putra, S. R., Moraes, F., & Hauff, C. (2018). SearchX: empowering collaborative search research. SIGIR '18: The 41st International ACM SIGIR Conference on Research & Development in Information Retrieval, 1265–1268. 10.1145/3209978.3210163

[CR84] Ramirez, A., & Walther, J. B. (2015). Information seeking and interpersonal outcomes using the internet. T. Afifi, & W. Afifi. Uncertainty, information management, and disclosure decisions. New York: Routledge, 10.4324/9780203933046.

[CR85] Reddy BS, Krishnamurthy M, Asundi A (2018). Information Use, User, User Needs and Seeking Behaviour: A Review. DESIDOC Journal of Library & Information Technology.

[CR86] Renner B, Prilla M, Cress U, Kimmerle J (2016). Effects of prompting in reflective learning tools: Findings from experimental field, lab, and online studies. Frontiers in Psychology.

[CR87] Resnick L, Asterhan C, Clarke S (2015). Socializing intelligence through academic talk and dialogue.

[CR88] Rieh SY, Kim YM, Markey K (2012). Amount of invested mental effort (AIME) in online searching. Information Processing and Management.

[CR89] Rieh SY, Collins-Thompson K, Hansen P, Lee H-J (2016). Towards searching as a learning process: A review of current perspectives and future directions. Journal of Information Science.

[CR90] Roschelle, J., & Teasley, S. D. (1995). The construction of shared knowledge in collaborative problem solving. In C. O’Malley (Ed.), *Computer supported collaborative learning* (pp. 69–97). Springer. 10.1007/978-3-642-85098-1_5.

[CR91] Rouet, J.-F., & Britt, M. A. (2011). Relevance processes in multiple document comprehension. In M. T. McCrudden, J. P. Magliano, & G. J. Schraw (Eds.), *Text relevance and learning from text* (pp. 19–52). IAP Information Age Publishing.

[CR92] Second author & first author (submitted). The role of collaborative argumentation in future teachers’ sourcing of online information. Submitted for review to the German Journal of Educational Psychology.

[CR93] Shah, C. (2009). Lessons and Challenges for Collaborative Information Seeking (CIS) Systems Developers. CIB workshop at GROUP 2009. Sanibel Island, Florida.

[CR94] Shah C, Woodsworth A (2010). Collaborative information seeking: A literature review. Advances in Librarianship (Advances in Librarianship, Vol. 32).

[CR95] Shah C (2014). Collaborative information seeking. Journal of the Association for Information Science and Technology.

[CR96] Shah C, Capra R, Hansen P (2017). Research agenda for social and collaborative information seeking. Library & Information Science Research.

[CR97] Shin D, Park YJ (2019). Role of fairness, accountability, and transparency in algorithmic affordance. Computers in Human Behavior.

[CR98] Siemon D, Becker F, Eckardt L, Robra-Bissantz S (2019). One for all and all for one - towards a framework for collaboration support systems. Education and Information Technology.

[CR99] Sillence E, Briggs P, Harris P, Fishwick L (2006). A framework for understanding trust factors in web-based health advice. International Journal of Human Computer Studies.

[CR100] Sinatra GM, Pintrich PR (2003). Intentional conceptual change.

[CR101] Solli A, Mäkitalo Å, Hillman T (2018). Rendering controversial socioscientific issues legible through digital mapping tools. International Journal of Computer-Supported Collaborative Learning.

[CR102] Spence, P. R., Reddy, M. C., & Hall, R. (2005). A survey of collaborative information seeking practices of academic researchers. *Proceedings of the 2005 International ACM SIGGROUP Conference on Supporting Group Work - GROUP ‘05*, 85. 10.1145/1099203.1099216.

[CR103] Springer L, Stanne ME, Donovan SS (1999). Effects of small-group learning on undergraduates in science, mathematics, engineering, and technology: A meta-analysis. Review of Educational Research.

[CR104] Sun, Y., & Kantro, P. B. (2006). Cross-Evaluation: A new model for information system evaluation. *Journal of the American Society for Information Science and Technology, 57*(5), 614–628. 10.1002/asi.20324

[CR105] Sundar SS, Metzger MJ, Flanagin AJ (2008). The MAIN model: A heuristic approach to understanding technology effects on credibility. Digital media, youth, and credibility.

[CR106] Tabak I, Corno L, Anderman EM (2015). Functional scientific literacy: Seeing the science within the words and across the web. Handbook of educational psychology.

[CR107] Teasley S, Resnick LB, Säljö R, Pontecorvo C, Burge B (1997). Talking about reasoning: How important is the peer in peer collaboration?. Discourse, tools and reasoning: Essays on situated cognition.

[CR108] Thiebach M, Mayweg-Paus E, Jucks R (2016). Better to agree or disagree? The role of critical questioning and elaboration in argumentative discourse. Zeitschrift Für Pädagogische Psychologie.

[CR109] Thomm E, Bromme R (2016). How source information shapes lay interpretations of science conflicts: Interplay between sourcing, conflict explanation, source evaluation, and claim evaluation. Reading and Writing.

[CR110] Tsai, M.-J., Hsu, C.-Y., & Tsai, C. -C. (2012). Investigation of high school students’ online science information searching performance : The role of implicit and explicit strategies. *Journal of Science Education and Technology, 21*, 246–254. 10.1007/s10956-011-9307-2.

[CR111] Twidale, M. B., Nichols, D. M., & Paice, C. D. (1997). Browsing is a collaborative process. *Information Processing & Management, 33*(6), 761–783. 10.1016/S0306-4573(97)00040-X

[CR112] Vogel F, Wecker C, Kollar I, Fischer F (2017). Socio-cognitive scaffolding with computer-supported collaboration scripts: A meta-analysis. Educational Psychology Review.

[CR113] von Aufschnaiter C, Erduran S, Osborne J, Simon S (2008). Arguing to learn and learning to argue: Case studies of how students’ argumentation relates to their scientific knowledge. Journal of Research in Science Teaching.

[CR114] Walton DN (1989). Dialogue theory for critical thinking. Argumentation.

[CR115] Wathen CN, Burkell J (2002). Believe it or not: Factors influencing credibility on the web. Journal of the Association for Information Science and Technology.

[CR116] Watts M, Alsop S, Gould G, Walsh A (1997). Prompting teachers’ constructive reflection: Pupils’ questions and critical incidents. International Journal of Science Education.

[CR117] Weinberger A, Fischer F (2006). A framework to analyze argumentative knowledge construction in computer-supported collaborative learning. Computers & Education.

[CR118] Wilson TD (2000). Human information behavior. Informing Science.

[CR119] Zhao Y, Zhang J (2017). Consumer health information seeking in social media: A literature review. Health Information and Libraries Journal.

[CR120] Zimmermann M, Jucks R (2018). How experts´ use of medical technical jargon in different types of online health forums affects perceived information credibility: A randomized experiment with laypersons. Journal of Medical Internet Research.

[CR121] Zimmermann, M., & Jucks, R. (2018b). With a view to the side: YouTube’s sidebar and YouTuber’s linguistic style as hints for trust-related evaluations. *International Journal of Human-Computer-Interaction.*10.1080/10777318.2018.1519165.

